# DNA-directed immobilization fluorescent immunoarray for multiplexed antibiotic residue determination in milk

**DOI:** 10.1007/s00216-024-05481-9

**Published:** 2024-08-28

**Authors:** J. Guercetti, N. Pascual, A. Aviñó, R. Eritja, J.-P. Salvador, M.-P. Marco

**Affiliations:** 1 Nanobiotechnology for Diagnostics (Nb4D), Department of Chemical and Biomolecular Nanotechnology, Institute for Advanced Chemistry of Catalonia (IQAC), Spanish Council for Scientific Research (CSIC), Jordi Girona 18-26, Barcelona, 08034 Spain; 2 CIBER de Bioingeniería, Biomateriales y Nanomedicina (CIBER-BBN), Madrid, Spain; 3 Nucleic Acid Chemistry Group, Department of Chemical and Biomolecular Nanotechnology, Institute of Advanced Chemistry of Catalonia (IQAC), Spanish National Research Council (CSIC), Jordi Girona 18-26, Barcelona, 08034 Spain

**Keywords:** DNA-directed immobilization, Microarray, Antibodies, Antibiotic

## Abstract

The presence of antibiotic residues in cow’s milk entails high risk for consumers, the dairy industry, and the environment. Therefore, the development of highly specific and sensitive screening tools for the rapid and cost-effective identification of traces of these compounds is urgently needed. A multiplexed screening platform utilizing DNA-directed immobilization (DDI) was developed aiming to detect three classes of antibiotic residues (fluoroquinolones, sulfonamides, and tylosin) prevalently found in milk. Throughout this work, each oligonucleotide sequence was conjugated to a different hapten molecule, while the three complementary strands were immobilized in 24 independent microarray chips on a single glass slide. First, the array was incubated with the pool of hapten-oligonucleotide conjugate site encoded the signal through DNA hybridization. Next, commercial milk samples were incubated with the cocktail of monoclonal antibodies following a secondary fluorophore-labeled antibody which was required for fluorescent readout. Direct sample detection was achieved in milk diluting 20 times in assay buffer. The limits of detection (LODs) reached were 1.43 µg kg^−1^, 1.67 µg kg^−1^, and 0.89 µg kg^−1^ for TYLA, STZ, and CIP, respectively, which represented in raw milk 7.15 µg kg^−1^, 8.35 µg kg^−1^, and 4.45 µg kg^−1^ for TYLA, STZ, and CIP, respectively, that are below the EU regulatory limits. Cross-reactivity profiles were evaluated against the family of structurally related antibiotics in order to demonstrate the capability to detect antibiotics from the same family of compounds. A pre-validation study was performed by spiking 20 blind samples above and below the maximum residue limits established by the EU guidelines. The system was successfully implemented towards randomized sample classification as compliant or non-compliant. The proposed DDI-based immunoarray provides a fast and cost-effective alternative to obtain semi-quantitative information about the presence of three veterinary residues simultaneously in milk samples.

**Graphical Abstract:**

Antibody fluorescent microarray based on DDI. The figure shows the main steps involved in the immunoassay. First, the printing of the oligo N4-6_down_ probe over the glass slide, followed by an incubation with a complementary strand conjugated to the hapten and finally the selective recognition using monoclonal antibodies and fluorescent quantification.

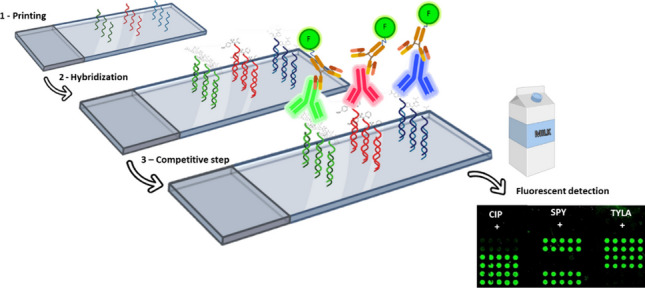

**Supplementary Information:**

The online version contains supplementary material available at 10.1007/s00216-024-05481-9.

## Introduction

Nowadays, antibiotics are the most effective chemical weapons available to deal with bacterial infections [1, 2]. The misuse of these natural or synthetic compounds during the last decades has been tackled as a major cause of antimicrobial resistance (AMR) worldwide [3]. According to the EARS-Net (European Antimicrobial Resistance Surveillance Network), around 800,000 infections from antimicrobial resistant bacteria were detected in the EU during 2020 and 35,000 people died as a consequence of infection [4]. Through the last years, AMR has reached concerning levels threatening public health systems, food industry, and environmental resources [5]. In the same line, multidrug-resistant strains are more frequently found and trends indicate a further increase in the forthcoming years if no alternative strategies are developed [6].

The pressure of selection towards resistant pathogens arises as a consequence of several factors, such as unprescribed consumption and administration of antibiotics as well as their overuse in food-producing animals or inadequate handling of residues contaminated with traces of these compounds [6, 7]. Alternative uses with prophylactic purposes or even as growth promoters have also been reported, although this practice is banned by legislation in the EU [8].

Antibiotic residues can be found in cow’s milk if the withdrawal periods are not strictly respected, imposing high risk for consumers as well as generating huge economic losses to the dairy industry [9]. According to the latest EFSA monitoring reports, the main groups of antibiotics in animal derivate products include β-lactams, tetracyclines, fluoroquinolones, sulfonamides, and macrolides [10]. In this regard, the European Commission has provided a set of guidelines that establish respective maximum residue limits (MRL) as the maximum allowed quantity (expressed in µg kg^−1^) of a potentially harmful compound present in food matrices for consumption and commercialization [11]. Additionally, the commission Decision 2003/181/EC, established minimum required performance limits (MRPLs) of analytical methods that aim to detect banned or prohibited substances in the EU community, applying to antibiotic residues as chloramphenicol or other compounds like nitrofurans metabolites. Different harmful substances such as pesticides are also regulated by the same determination of MRLs and MRPL undelaying tolerance levels food matrixes.

According to European directives, confirmatory and screening monitoring techniques are available in the food safety scenario. On the one hand, confirmatory methods should determine the exact composition of a sample, but require expensive equipment and trained personnel and are generally time-consuming [12, 13]. Currently, several protocols based on chromatographic techniques have been optimized to simultaneously screen the presence of these substances in milk; however, portable and easier to use alternatives are still required [14]. On the other hand, screening methods arise as promising tools for rapid and cost-effective sample characterization providing on-site and high-throughput analysis. Most common approaches include optical or colorimetric methods such as lateral flow immunoassays or similar configurations [15]. Nowadays, microbiological inhibition assays are widely applied as screening methods to detect antibiotics in milk due to their robustness and affordable price. Some drawbacks rely on the long incubation periods required (from 5 to 48 h) as well as controlled growing conditions [16]. In the last years, immunoassays have also gained attention due to the selective biorecognition properties. Antibodies are considered excellent bioreceptors to implement in sensing devices due to their high sensitivity, specificity, and robust performance in complex matrixes. Main limitations arise from their expensive and time-consuming production. Even though, the development of antibodies against small molecules (antibiotics, pesticides, or mycotoxins) entails a challenging task from an immunological perspective [17]. Several bioanalytical solutions targeting antibiotic residues in milk have been reported such as ELISAs [18], lateral flow immunoassays (LFIA) [19–21], or even biosensors coupled to optical [22, 23] and electrochemical transducers [24, 25]. However, multiplexation is still pointed as a major limitation over screening platforms due to the complexity behind signal interpretation [26] and possible non-specific interactions of reagents [27], while significant advantages arise including time and assay-cost savings or reduction in sample volume and reagents required [28]. In this regard, DDI and microarray technology emerge as highly compatible tools to expand multiplexed detection with site encoding response [29].

DDI consist of addressing biomolecules (small molecules, enzymes, antibodies, aptamers) previously conjugated to oligonucleotides, to their counterparts immobilized over a solid support (glass slide, gold layer, or even metal nanoparticles) through specific base pairing [29]. The concept and some applications of DDI were originally proposed by Niemeyer et al*.* [30] to create regenerable surfaces and give specific orientation to biomolecules and favoring multiplexation. A careful design and similar GC (%) content are required along the oligonucleotide sequence preparation to avoid possible interferences [31]. The performance of DDI was compared with direct spotting of antibodies and biotinylated antibodies over streptavidin-coated surfaces demonstrating that direct printing and DDI offer higher fluorescence reaching comparable limits of detection. Furthermore, DDI provided the best spot homogeneity with intra- and inter-experimental reproducibility with the added value of potential surface regeneration [31].

In previous reports, DDI was implemented towards the detection of anabolic androgenic steroids with success in our group [32, 33], demonstrating the feasibility of this multiplex platform. Thus, we propose the development of a multiplexed screening immunoassay with fluorescent readout to selectively discriminate between three main families of antibiotic residues found in cow’s milk samples. The system allows to process 24 samples simultaneously in less than 90 min to facilitate the decision-making process at production levels in dairy farms and industries.

## Materials and methods

### Reagents and immunoreagents

The immunoreagents used in this work for sulfonamides (SAs) and fluoroquinolones (FQs) detection were gently provided by Unisensor (Ougrée, Belgium) and described in previous reports [34, 35]. Tylosin’s immunoreagents were produced in our facilities and will be published elsewhere. The preparation of the BSA conjugates and the production of monoclonal antibodies (MAbs) have been performed with the support of the U2 of the ICTS “NANBIOSIS,” by the Custom Antibody Service (CAbS, CIBER-BBN, IQAC-CSIC). The rest of immunochemical reagents were purchased from Sigma-Aldrich (St. Louis, MO, USA). Oligonucleotides (N4_down/up_-NH_2_, N5_down/up_-NH_2_, and N6_down/up_-NH_2_) were acquired from Merck (Merck KGaA, Darmstadt, Germany) and the hapten-oligonucleotide conjugation (FQ: PrEDA-N4_up_, SA: SA2-N5_up_, and Tyl: hTB-N6_up_) will be described below. For the oligonucleotide conjugation, N,N′-dicyclohexylcarbodiimide 99% (DCC) and N-hydroxysuccinimide (NHS) were purchased from Sigma-Aldrich (St. Louis, MO, USA), and dimethyl pimelimidate (DMP) from Thermo Fisher Scientific (MA, USA) and the working aliquots were prepared in anhydrous dimethylformamide (DMF).

The target analytes under study, sulfapyridine (SPY), sulfatiazole (STZ), sulfametoxypiridazine (SMPZ), sulfatchloropiridazine (SCPZ), sulfadimetoxine (SDMX), sulfamethoxine (SMX), sulfamerazine (SRZ), sulfachinoxaline (SCX), sulfisomidine (SSD), enrofloxacin (ENFX), ofloxacin (OFX), ciprofloxacin (CPX), sarafloxacin (SFX), flumequin (FLUM), danofloxacin (DNFX), difloxacin (DFX), norfloxacin (NFX), tylosin A (TYLA), and tylosin B (TYLB) were delivered from Sigma-Aldrich and Honeywell. Sulfonamides and tylosin’s stock solutions were prepared at 10 mM concentration in DMSO, but quinolones were dissolved in 0.1 M NaOH. All of them were stored at 4 °C for a maximum period of 1 month. The secondary antibody, anti-mouse IgG-TRITC, was purchased from Sigma-Aldrich (St. Louis, MO, USA). Commercial milk samples were lyophilized and stored at − 40 °C, and once needed were resuspended with H_2_O ultrapure by stirring at 40 °C, considering density = 124.8 g L^−1^. Acetonitrile (ACN, HPLC gradient grade) was obtained from Panreac Quimica (Barcelona, Spain).

### Buffers

PBS is 0.01 M phosphate buffer (1.48 mM KH_2_PO_4_ and 8.3 mM Na_2_HPO_4_) in a 0.8% saline solution (137 mmol·L^−1^ NaCl, 2.7 mmol·L^−1^ KCl), at pH 7.5. PBST is PBS previously described with 0.05% Tween 20. The solution of PBST-Ca^2+^ corresponds to a 10 mM PBST pH 7.5 solution that contains 1 mM CaCl_2_. The printing buffer utilized for the deposition of oligonucleotides over the slide consisted of 150 mM di-sodium hydrogen phosphate (pH 8.5)/0.01% sodium dodecyl sulphate in H_2_O ultrapure. Hybridization buffer is composed of 10 mM Tris, 1 mM EDTA, and 1 M NaCl (pH 7.2). Borate buffer is 0.2 M boric acid/sodium borate (pH 8.7). Coating buffer is a 0.05 M carbonate-bicarbonate buffer (pH 9.6). Citrate buffer is 0.04 M sodium citrate (pH 5.5). For SPR experiments, the immobilization buffer was 10 mM sodium acetate (pH 4.0). Regeneration solutions consisted of 0.1 M glycine–HCL (pH 2.7) and 1 mM NaOH. The pH and the conductivity of all buffers and solutions were measured with the pH meter SevenCompact™ Duo S213 (Mettler Toledo, Spain).

### Oligonucelotide-hapten conjugation and characterization

The design of three oligonucleotide pairs (N4_down/up_, N5_down/up_, and N6_down/up_) was initially carried out. Through this work, each oligonucleotide pair will be identified with a number (4, 5, or 6) and the corresponding strand with the -up (conjugated to the hapten) or -down (immobilized in the glass surface) label. A similar G/C content, of around 50% (purines/pyrimidines), was considered during sequence generation. The three -down sequences (N4-6_down_) were constituted by 20 nucleotides and a carbon chain spacer of six atoms with an amino group (-NH_2_) in the 5′ end for their covalent attachment to the epoxy-silanized glass surface. Furthermore, similar criteria were used for the design of the upper oligonucleotides N4-6_up_, addressed to be linked to the antibiotic-hapten molecules through their carboxylic or amino groups.

#### Preparation of oligo-hapten conjugate N4_up_-PrEDA

The N4_up_-NH2 oligonucleotide was dissolved in 25 µL of ultrapure water and mixed immediately with 75 µL of 0.2 M sodium borate (pH 8.6). In parallel, PrEDA hapten (1 mg) and DMP (1 mg) were dissolved in hot anhydrous DMF (100 µL) and 0.2 mL 0.2 M sodium borate (pH 8.6), respectively. At the same time, 40 µL of PrEDA and 5 µL of DMP solutions were added immediately to the dissolved oligonucleotide. The whole mixture was stirred overnight at room temperature. Finally, the purification of the resulting hapten-oligo conjugate was carried out using NAP™-5 Columns Sephadex™ G-25 DNA grade columns from G&E HealthCare, eluted in water. Further purification was required by HPLC. HPLC conditions: Nucleosil 120–10 C18 column (250 × 4 mm). Solvent A: 5% ACN in 0.1 M aqueous TEAAc (triethylammonium acetate) (pH = 7) and solvent B: 70% ACN in 0.1 M aqueous TEAA (pH = 7). Flow rate: 1 mL/min. Conditions: 20 min linear gradient from 0 to 50% B. For the characterization of the conjugates, matrix-assisted laser desorption ionization time-of-flight (MALDI-TOF) was used on the products of oligo-hapten reaction. The conjugates were evaluated in a Bruker Autoflex III Smartbeam spectrometer (Billerica, MA). For this purpose, a mixture of 3-hidroxypicolinic acid (3 HPA)/ACN (1% V/V) 50 mg mL^−1^ and a solution of diammonium hydrogen citrate (AHC) (100 μg mL^−1^) were prepared (10:1 v/v, HPA: AHC) and 1 µL applied onto the target plate. Once dried, 1 μL of solution containing the oligonucleotide purified (non-conjugated sequence and the product of reaction) in water at concentrations ranging from (0.25 pmol µL^−1^ to 5 pmol µL^−1^) the mixture 1:1:1 of THAP:CA:SAMPLE completely dry drop takes 25 min. Methods: LN_ProtMix.Par, Detection 3000–10,000 Da, Processing: SC_Protein_Low, Laser Power: 95%. MALDI-TOF m/z (negative mode) N4_up_-PrEDA, calc 6256.21, found 6812.62.

#### N5_up-_SA2 and N6_up_-hTB oligo-hapten conjugate

The oligos (N5_up_ and N6_up_) were dissolved in 0.2 mL of ultrapure water and mixed with 0.1 mL of 0.2 M sodium borate (pH 8.6). In this case, SA2 and hTB were activated by active ester method that consisted of dissolving (10 µmols) each of them in 100 µL of DMF and mixed with 50 µL of NHS (25 µmols) and 50 µL of DCC (50 µmols). The mixture was left with magnetic stirring for 3 h at room temperature, until the solution became opaque due to the precipitation of the urea. Then, we centrifuge (10,000 rpm for 15 min) to remove the precipitate and the supernatant was added to the corresponding aqueous solution of the amino-oligonucleotide. The mixture was left stirring and kept o.n. at 4 °C. The mixture was purified and characterized as described above. MALDI-TOF m/z (negative mode) N5_up_-SA2, calc 6256.21, found 6532.02 and N6_up_-hTB, calc 6256.21, found 7086.60.

### Fluorescent DDI microarray

#### DDI array printing conditions

Plain glass slides (75 × 25 mm) purchased from Corning Inc (Corning, NY, USA) were cleaned and derivatized with epoxy groups following the standard protocol already described by Sanchis [36]. Afterwards, the lower oligonucleotides N4-6_down_ were covalently deposited onto the glass surface through the synthesized 5′-NH_2_ end. Probe immobilization was performed using an automated piezo-driven SciFlexArrayer S3 (Scienion AG, Berlin, Germany). Drop deposition was carried out using a piezo dispense capillary (PDC) 70 type 1, setting the voltage and the pulse width at 98 V and 50 µs respectively. For the array construction, 5 drops (350 pL each) were deposited per spot and the temperature set at 25 °C with humidity at 65%, allowing slides to dry for 1 h after printing process in the spotting chamber and then kept at 4 °C until use for a maximum of 5 days. Each final array configuration is constituted by a 5-column × 6-row matrix (dedicating 2 rows with 5 replicate spots, per oligonucleotide N_down_). In addition, 24 identical arrays were printed in a single glass slide to match with the ArrayIt® holder (ArrayIt® Corp, Sunnyvale, USA) utilized to perform the assays. This gasket allows the generation of independent wells over the same slide for simultaneous determinations.

#### Monoplexed DDI microrarray

In the case of single assays following an indirect competitive format, individual calibration curves in PBST-Ca^2+^ and 1/5 milk dilution in the same buffer were generated within a range of 5 µM to 0.5 nM. Ten replicate spots of each oligo N4-6_down_ solution were deposited in separate sub-arrays. For this, optimized spotting concentrations were established towards each oligonucleotide strain N4_down_ at 75 μg mL^−1^, N5_down_ at 50 μg mL^−1^, and N6_down_ at 6.25 μg mL^−1^ in printing buffer. After optimization experiments, assessing different immobilization concentrations of upper and lower oligos, the most suitable pair concentration was selected for each system under monoplex format. Initially, a washing step with 10 mM PBST was performed followed by the incubation with the corresponding complementary chains at 0.1 μg mL^−1^ for the three oligonucleotide hapten conjugates (N4_up-_PrEDA 0.1 μg mL^−1^, N5_up-_SA2 0.1 μg mL^−1^, N6_up-_hTB 0.1 μg mL^−1^). After 30 min of incubation, a washing step (3 × 200 μL PBST) was required to proceed to the competitive step incubating the analyte with the respective monoclonal antibody at 1 μg mL^−1^ MAb-FQ, 0.25 μg mL^−1^ MAb-SA, and 0.06 μg mL^−1^ MAb-Tyl. Then after additional 30 min, a washing step was performed and the array was incubated with a secondary Anti-Mouse-IgG-TRITC antibody (Abcam, England) at 1/250 in PBST 10 mM. A final wash with PBST and ultrapure water was required, and then, slides were dried with N_2_ stream.

#### Multiplexed DDI microarray

After printing, a washing step (3 × 200 µL PBST) was carried out, and afterwards, the chips were incubated with 100 µL of the “pool of oligonucleotides” constituted by a mixture of the three oligo-hapten conjugates (oligo N4-6_up_) at standardized assay concentrations (N4_up_-PrEDA 1 μg mL^−1^, N5_up_-SA2 0.5 μg mL^−1^, and N6_up_-hTB at 0.2 μg mL^−1^ in hybridization buffer). After 30 min, a washing step (3 × 200 µL PBST) took place and the “Cocktail of monoclonal antibodies” (50 µL/well) defined by the combination of the three MAbs (MAb-FQ in a final concentration 0.8 μg mL^−1^, MAb-SA in a concentration 0.6 μg mL^−1^, and MAb-Tyl in a concentration of 0.42 μg mL^−1^ in 10 mM PBST and 1 mM Ca^2+^) with different analyte concentrations ranging from 0.5 nM to 5 µM (50 µL/well) (CIP, STZ, TYLA) in buffer or milk diluted 1/20 PBST Ca^2+^ for the competitive step during 30 min. After another washing step, the incorporation of the secondary labeled Anti-mouse-IgG-TRITC antibody at 1/250 in 10 mM PBST for 30 min was done under stirring conditions (350 rpm) and covered from light. Finally, a washing step (3 × 200 µL of PBST) and 1 × 200 µL in ultrapure water were required to then dry the slides with N_2_ and continue with the fluorescent readout.

#### Signal acquisition

Microarray measurements were acquired in a dual laser microarray scanner InnoScan 710 (Innopsys, Carbonne, France) with an optical filter with 10-µm resolution using 532-nm laser excitation wavelength. The laser power and photomultiplier tube (PMT) gain were set to 95% and 80%, respectively. The spots were measured by deducting the mean TRITC background intensity to the mean of TRITC foreground intensity using Mapix—Microarray image acquisition and analysis software (Innopsys, Carbonne, France). Calibration curves were estimated from the average fluorescence intensity of ten replicate spots for each bioconjugate expressed in RFU (relative fluorescence units). The respective curves were fitted to a four-parameter logistic equation using the software GraphPad Prism V.7 (GraphPad Software Inc., San Diego, CA, USA) according to the formula: *Y* = [(*A* − *B*)/1 − (*x*/*C*)*D*] + *B*, where *A* is the maximal fluorescence, *B* the minimum fluorescence, *C* the concentration producing 50% of the difference between *A* and *B* (or IC_50_), and *D* the slope at the inflection point of the sigmoid curve. The limit of detection (LOD) was defined as the concentration producing 90% of the maximal fluorescence (IC_90_).

### Multianalyte immunoarray profile

Standards solutions for the three reference antibiotics selected and structurally related compounds were prepared at three different concentrations. Working solutions were prepared at the MRL concentration for the corresponding target compound, 2 times the MRL, and 0.5 the MRL value in milk diluted 1/20 in PBST Ca^2+^. Different sulfonamides, fluoroquinolones, and macrolides were analyzed. After the addition of the target analyte, all antibodies were incorporated and incubated for 30 min at RT. The standard assay procedure was then carried out.

### Platform pre-validation

Commercial milk samples were spiked at different concentrations above and below the reference MRL established for each antibiotic and were randomly assigned in each well. For the assay, 5 μL of spiked milk was diluted in 95 μL of 10 mM PBST Ca^2+^ over each well in blind conditions following the assay protocol previously described. Moreover, negative (zero concentration) and positive control solutions (spiked at MRL value for each reference analyte) were also measured on the same slide to define the threshold value for positive or negative sample. The intensity obtained from negative samples (zero samples) was normalized and referenced as 100% of the signal and fluorescence from samples doped at MRL level was used to define the cutoff value to consider either positive or negative antibiotic levels according to regulatory limits.

## Results and discussion

Every year, the European Food Safety Agency (EFSA) reported the results of non-compliant or suspect samples that are contaminated with the priority target compounds. Among all of them, antibiotics (group B1) are listed and found in different matrices which included dairy products such as milk. In the latest reports from EFSA [37], non-compliant milk samples for penicillins were the most detected antibiotics although others such as tetracyclines were also found. However, other antibiotics may be present taking into consideration their high amount produced for animal treatment that involves penicillins, tetracyclines, and also sulfonamides, fluoroquinolones, and macrolides [37]. In respect to EC third-producing countries, different reports identified that the presence of a wide antibiotic family compound was detected in milk samples [38, 39]. In this regard, the regulation (EU) No. 37/2010 laid down the maximum residue limits allowed for antibiotic residues in milk. Taking into consideration the prevalence of the main antibiotics that may be found in milk, we chose to face this work for fluoroquinolones, sulfonamides, and tylosines antibiotics to be detected.

### Immunochemical characterization of the monoclonal antibodies

Antibodies employed throughout this work are considered “class-selective antibodies,” and cross-react selectively with structural analogues but not with other antibiotic families. For instance, the antibody against sulfonamides [34] is able to bind with different affinity to a broad range of sulfonamides that share a common structural epitope without interacting with fluoroquinolones or macrolides. The IC50 reached using those polyclonal antibodies was 2.86 ± 0.24 µg·L^−1^ in terms of sulfapyridine antibiotic in buffer. In the case of FQ assays, the initial immunoreagent characterization performed by ELISA took place using enrofloxacin as reference analyte due to a better sensibility described under this format. However, for microarray experiments, the reference analyte was changed for CIP considering reports evidencing that CIP was the most abundant metabolite of cow prescribed with enrofloxacin [40] assuming a prevalence of this compound in real samples. Additionally, this particular assay requires the incorporation of Ca^2+^ in the buffer because the PrEDA hapten and the target FQ analytes are able to chelate divalent cations such as Ca^2+^ [41]. Therefore, the antibodies produced recognize with higher affinity the hapten structure and the analyte complexed to divalent cations as it was demonstrated by Pinacho et al*.* [42] who reached an IC50 of 0.35 μg L^−1^ for ciprofloxacin antibiotic in buffer.

Initially, single analyte ELISAs (see Electronic Supplementary Material Fig. S1 and Table S1 from supporting information) were performed prior to the implementation under DDI-based microarray format. The selection of the most suitable concentrations of BSA conjugate and antibody was accordingly performed by 2d-checkerboard titration well established in our laboratory. Afterwards, the calibration curves were constructed in order to know the detectability of the ELISA assay. The IC50 obtained for each calibration curve was 3.83 ± 0.03, 5.56 ± 0.08, and 2.78 ± 0.06 μg L^−1^ for enrofloxacin (PrEDA-BSA vs MAb-FQ), sulfapyridine (SA2-BSA/MAb-SA), and tylosine B (hTB-BSA/MAb-Tyl), respectively. In general, IC50 is used as a characteristic parameter to evaluate the detectability of the developed assays. Thus, the analytical parameters reached were comparable among the previous work published working with polyclonal antibodies [34, 42].

Further antibody characterization was the evaluation of kinetic parameters and affinity constant determination of the three monoclonal antibodies and was carried out using SPR technique with BiacoreT200 system (see Electronic Supplementary Material Fig. S2 and Table S5). KD is the affinity constant and is a characteristic parameter for the affinity of the monoclonal antibodies. According to our results, the reagents with stronger affinity were MAb-Tyl with BSA-hTB conjugate reaching a KD of 0.136 nM, followed by MAb-FQ and BSA-PrEDA with a KD of 7.14 nM and in third place the MAb-SA for BSA-SA2 with a KD of 10.98 nM differing in one order of magnitude respectively. These experiments were performed in PBST-Ca^2+^ as running buffer required for the FQ’s assay and also contemplating that would be implemented as assay buffer in the final multiplexed format. The results revealed a highest affinity of MAb-Tyl according to the high homology between the hTB and the hTA which was used as an immunogen for the production of MAb-Tyl. For MAb-FQ and MAb-SAs, the KD obtained were similar, in the range of nM. The interaction was lower compared with the MAb-Tyl because the heterology degree is higher between the haptens used as a competitor for FQ and SA.

### Oligo-hapten conjugation

**Fig. 1:**
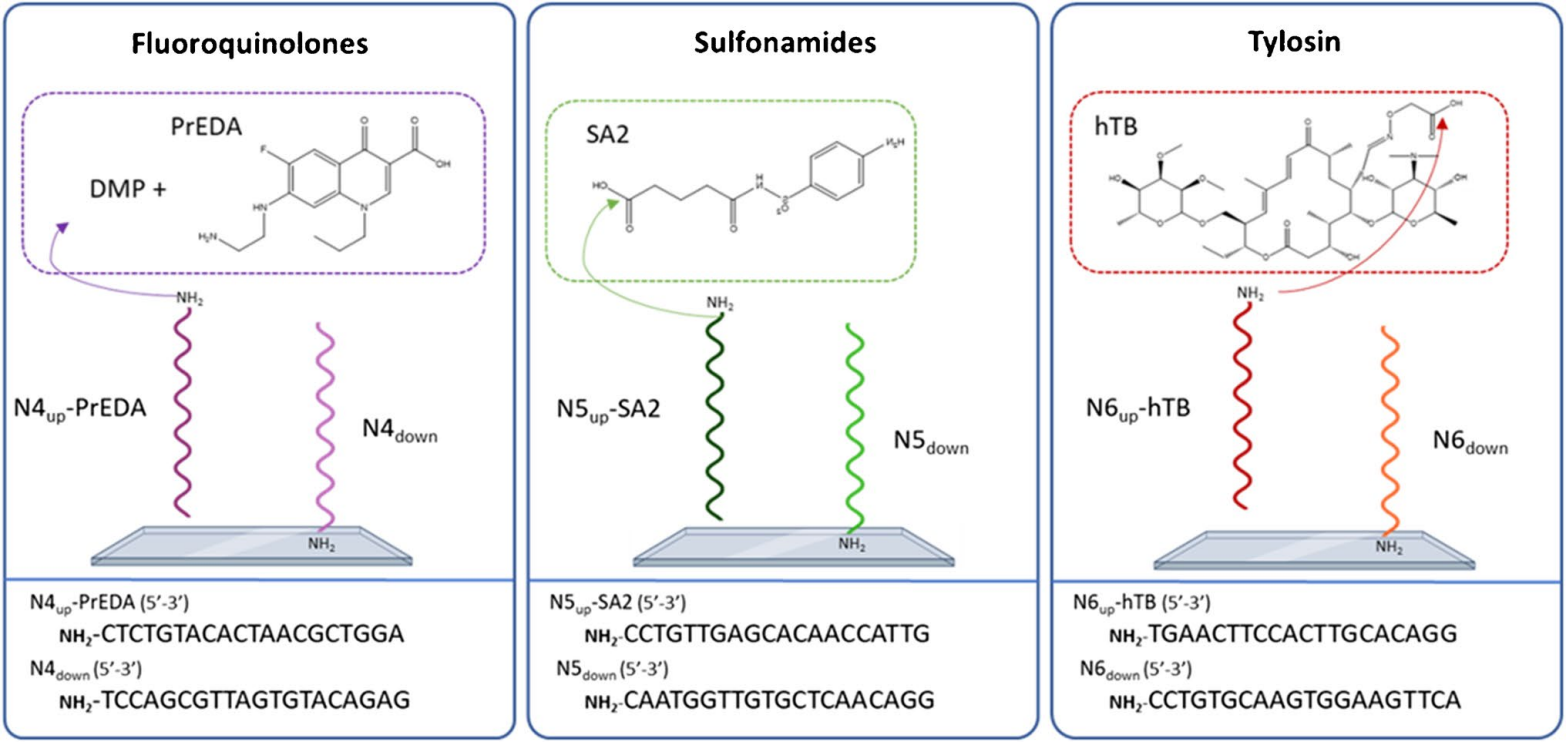
Schematic representation of the oligonucleotide sequences used for DDI immobilization and the conjugation of the corresponding haptens for the three types of antibiotic families (FQs, SAs, and TYLs). The spatial distribution of the oligonucleotide probes on the DDI microarray is also described

Oligonucleotide strains were synthesized containing a carbon spacer of 6 atoms and an amino group in the 5′ end to covalently bind the oligos N4-6_down_ to the glass surface and for the case of oligo N4-6_up_, as coupling moiety to corresponding antibiotic derived haptens that will serve as competitors in the immunoassay as displayed in Fig. 1. The corresponding oligos N4-6_up_ were successfully conjugated to the haptens (PrEDA, SA2, and hTB, respectively) according to the MALDI-TOF MS data obtained. All conjugates were purified by HPLC and characterized by MALDI-TOF–MS prior to their implementation in the fluorescent DDI array.

### Fluorescent DDI microarray establishment

One of the most critical steps in developing competitive immunochemical assays relies on the careful selection of the antibody concentration and its competitor to reach sufficient sensitivity and maximum signal. Particularly in the case of DDI, the optimization of oligo N4-6_down_, N4-6_up_-hapten conjugate, and monoclonal antibodies was required. For this, three-dimensional studies exploring the optimum coating concentration of oligos N4-6_down_ were assessed dispensing 5 drops per spot (matrix 4 × 5) of the corresponding concentration of 200, 100, 50, and 25 μg mL^−1^. Simultaneously, the addition of different concentrations of upper oligo N4-6_up_ was also evaluated by incubating 0.1 μg mL^−1^, 1 μg mL^−1^, and 10 μg mL^−1^ of oligo-hapten conjugate solutions over the spotted array. Finally, the optimization of MAb required for each analyte was considered studying the maximum signal obtained for a given concentration of analyte established ideally between 10,000 and 20,000 RFUs in PBST Ca^2+^ buffer. According to this experiment, we ensure the complete hybridization of N_down_/N_up_-hapten pair, and then, we selected the appropriate concentration of N_down_/N_up_-hapten and MAb for a competitive assay which is stated in between 70 and 80% of the saturation curve.

**Fig. 2:**
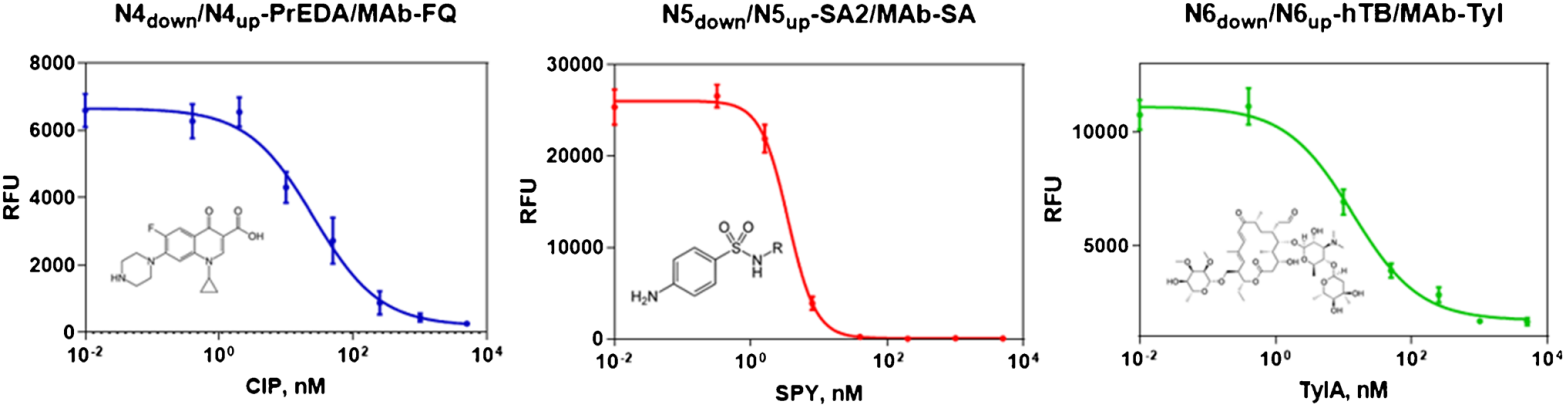
Single analyte indirect competitive DDI microarray in PBST Ca^2+^. Calibration curves were obtained for the selected target analytes to estimate the analytical performance of the immunoassay in buffer. A matrix of 2 × 5 was printed in individual sub-arrays, with a total of ten replicate spots for each oligonucleotide pair. The SD corresponds to the value defined with 2 independent wells

Single analyte detection in an indirect competitive format was achieved throughout the generation of calibration curves for TYLA, SPY, and CIP in PBST Ca^2+^ (see Fig. 2 and Table 1). In the case of CIP detection, the LOD reached with the individual DDI-based arrays was 2.17 µg L^−1^ far below the MRL established at 100 µg kg^−1^. On the other hand, the limit of detection obtained for TYLA was 1.26 µg L^−1^ while SPY was detectable at 1.03 µg L^−1^. Therefore, the DDI-based approach proposed was successfully implemented in the detection of the three target analytes individually with comparable performance to the ELISA reference method.

**Table 1:** Comparison of assay performance under single and multiplex format in buffer and matrix. The optimum conditions were set for each assay considering the final application in multiplexed format

DDI assay features	FQ		SA^b^		TYL	
	Monoplex Buffer	Multiplex Milk 1/20	Monoplex Buffer	Multiplex Milk 1/20	Monoplex Buffer	Multiplex Milk 1/20
RFU_max_	4818 ± 167	22,884 ± 984	30,755 ± 1211	20,269 ± 997	15,017 ± 217.3	20,515 ± 878
RFU_min_	526.3 ± 201	1033 ± 254	382.5 ± 103	210.2 ± 114	355.5 ± 130	690.2 ± 120
Slope	− 1.41 ± 0.41	− 1.27 ± 0.24	− 0.87 ± 0.12	− 1.38 ± 0.32	− 0.82 ± 0.04	− 1.72 ± 0.13
IC50^a^	4.08 ± 0.18	1.91 ± 0.83	5.43 ± 1.78	2.43 ± 0.23	13.56 ± 3.56	4.79 ± 0.92
LOD^a^	2.17 ± 0.44	0.89 ± 0.54	1.03 ± 0.87	1.67 ± 0.40	1.26 ± 1.02	1.43 ± 0.62
*R*^2^	0.99 ± 0.01	0.96 ± 0.09	0.99 ± 0.01	0.98 ± 0.01	0.99 ± 0.01	0.99 ± 0.01

^a^The concentrations are expressed in µg L^−1^. ^b^SA’s monoplexed assays were carried out using SPY as reference, and multiplex assay STZ was used as reference target once the profile analysis study was conducted. The SD was estimated from two different wells

#### Matrix effect of DDI array

The assay performance in diluted milk (1/5) was evaluated prior to the implementation in real samples. For this purpose, calibration curves in buffer (PBST Ca^2+^) and in milk diluted 5 times in the same buffer were generated for the three reference analytes. A comparable analytical performance was obtained when the assay was conducted in milk 1/5 or directly in PBST-Ca^2+^ when we compare the DDI array versus the ELISA (see Electronic Supplementary Material Fig. S1) and no considerable differences in the detectability were observed in respect to buffer conditions. From Fig. 2, the maximum signal (RFU_max_) only decreased in CIP assay using milk diluted 1/5 but the analytical parameters are comparable showing adequate analytical performance. The other two analytes (SPY and TYLA) were detectable in PBST and milk 1/5 without considerable differences.

Thus, a single and common sample dilution was established for the three assays in order to detect each analyte (based on the MRL) in a suitable working range. For this purpose, a 20-fold dilution of commercial milk was carried out in PBST Ca^2+^ prior to the incorporation in the multiplexed assay. According to the studies performed in ELISA, a similar behavior is expected to have between 1/5 and 1/20 milk dilution factor with the DDI multiplexed format (see Table 1). In addition to this, sulfonamide assay showed high sensitivity in respect to the MRL (100 μg kg^−1^); therefore, such dilution was required to discriminate between compliant and non-compliant samples in the working range.

#### Shared- and cross-reactivity phenomena assessment

**Fig. 3:**
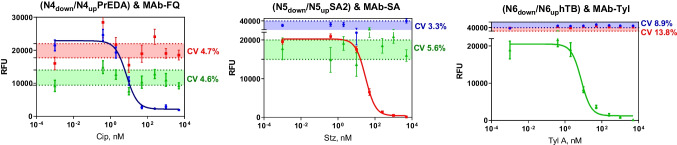
Cooperative phenomena studies. Single analyte calibration curves using the pool of haptenized oligos and the cocktail of MAbs for each analyte, showing the CV % of the maximum signal detected from non-specific reagents in each assay. Each data point in the graphs corresponds to 5 replicate spots inside each microarray chip. Analytical performance of the assays was plotted in the tables, extracted from the logistic equation used to fit the standard curve. The results shown correspond to the average and standard deviation of two different wells and the schematic representation of the reagents behavior in each well is plotted. ^a^IC50 expressed in µg kg.^−1^

In order to elucidate the specificity provided by DNA hybridization reaction, the selectivity of the monoclonal antibodies for their respective analytes, and the specificity against their corresponding hapten-oligonucleotide conjugate, cross- and shared-reactivity studies were carried out, respectively. Shared-reactivity was assessed by studying how the selective antibodies will bind the haptens already immobilized in the glass surface through DDI. As it can be observed in Electronic Supplementary Material Fig. S4 from supporting information, no interaction was shown for the specific antibodies against the other haptens immobilized in the same well, so any shared- and cross-reactivity is shown. Thus, the cocktail of antibodies can be used for detecting the corresponding target analyte. As evidenced in Fig. 3, each target analyte is selectively recognized although the cocktail of antibodies is utilized. In the range of concentration where the target analyte was tested, any cross-reactivity was evidenced, obtaining the highest fluorescent response in other assays when the target analyte was not present. Hence, these results encourage the use of DDI for multiplexed screening purposes. According to the data extracted from Fig. 3, the coefficient of variation (CV%) in the maximum signal (zero analyte) over nonselective reagents for each assay was calculated to corroborate the absence of potential interferences at increasing analyte concentrations combining the set of upper oligonucleotides and MAbs. For FQ multiplexed assay, the CV for SA’s reagents was 4.7% and TYL 4.6% demonstrating specific response. Similar behavior was observed in the case of SA’s assay reaching CVs of 5.6% for TYL and 3.3% for FQ reagents. While at increasing concentrations of TYLA, the signal fluctuations over the other reagents included in the same well were 8.9% for FQ and 13.8% for SA’s reagents.

#### Multiplexed assay sensitivity

Lack of shared- and cross-reactivity with the immunoreagents under multiplexed format was demonstrated (see Fig. 3). The assay sensitivity was calculated using the criteria which the limit of detection (LOD) is defined as the IC90 (10% of the maximum signal). This criterion is well established in our laboratory and used by other groups in the immunochemistry field [43–45]. Thus, the assays were evaluated reaching limits of detection in diluted matrix 1/20 of 0.89 µg kg^−1^ for CIP, 1.67 µg kg^−1^ for STZ, and 1.43 µg kg^−1^ for TYLA. However, the real concentration in undiluted matrix is 20 times higher, due to the sample dilution required. Baring this in mind, the minimum amount detected for each assay in direct matrix would be 17.8 µg kg^−1^ for CIP, 33.4 µg kg^−1^ for STZ, and 28.6 µg kg^−1^, all of them below the MRL established by regulatory authorities. These findings highlight that multianalyte determination of three target analytes was successfully achieved committing to EU guidelines. Moreover, we have conducted reproducibility studies to evaluate the variability inter-day for IC50 for each corresponding analyte; we have found a CV of 11.9, 32.7, and 51.5% for CIP, SPY, and TYLA, respectively. Thus, a positive control will be included in the microarray for each family of antibiotics as a decision fluorescent point to discriminate between compliant and non-compliant samples.

### Reactivity profile analysis

**Fig. 4:**
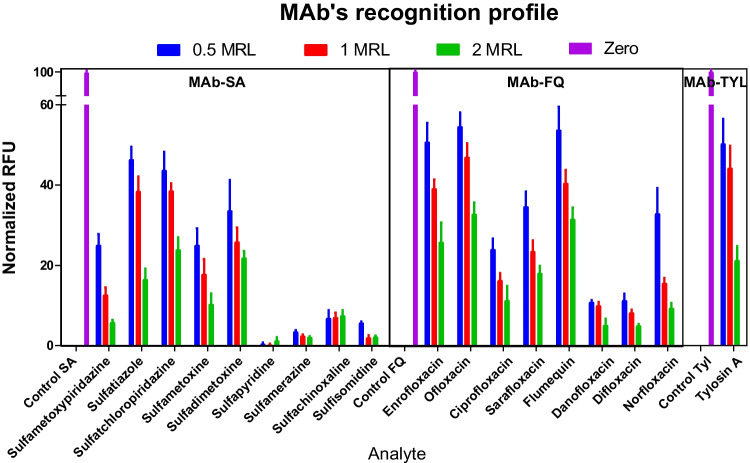
Reactivity profile assessment. The detection of family of compounds at the level of the MRL was evaluated showing the potential application in the real field. Milk samples were spiked at the level of interest (MRL) and above and below (2 times MRL or 0.5 MRL) and then analyzed with the DDI-based microarray. Each assay was normalized considering 100% to the maximum signal obtained for the sample with zero content of analyte. A competitive assay format inversely correlates signal value with concentration. The SD reported corresponds to 10 replicate spots per analyte assessed

Considering the preference of a single sample dilution for the three assays and the use of class-selective antibodies, the selection of the most adequate reference target analyte was required to be evaluated. In this regard, the reactivity profile (see Fig. 4) of each MAb was studied against other structurally related antibiotics under multiplexed configuration. A total of 9 SAs, 8 FQs, and 2 Tyls were measured to find the less sensitive analogues to be implemented as reference in each assay, also considering previous reports in ELISA [34, 35]. By selecting the reference analytes with worst detectability, it was expected to identify all the family of compounds at the level of interest (MRL) in a semi-quantitative approach, extending the detection of three analytes over more than 18 different molecules. From this experiment, STZ was selected as reference analyte for sulfonamide assay due to the lower detectability achieved in comparison with SPY, initially utilized for platform characterization experiments. The same study was applied over FQs, finding that ciprofloxacin was the best candidate as target analyte also allowing the detection of the rest of structural analogues. In the case of macrolides, the antibody (MAb-Tyl) was selective towards TYLA and TYLB, using the first as reference.

### Pre-validation in real milk samples

In order to assess the performance of the DDI chip, a pre-validation study was conducted in blind conditions. For this purpose, 20 commercial milk samples were spiked at different levels above and below the MRLs established for each analyte and diluted in the assay buffer 20 times. The suspected samples were prepared in blind conditions including a negative control (zero concentration) and also MRL controls (spiked at the level of interest for the three analytes) in the same slide. Then, the samples were randomly distributed in the wells; semi-quantitative results were obtained after fluorescent readout.

Following the indirect assay format, a sample was classified as suspected positive if the signal obtained was lower than the MRL value and, on the contrary, a negative sample was evidenced with higher fluorescence respecting the MRL value and lower than the zero. Relative fluorescence unit (RFU) values were normalized and the graph is expressed in normalized RFUs to avoid differences in the maximum response among the three immunoassays. Throughout, this approach is possible to perform a semi-quantitative analysis by referring the signal obtained with the unknown sample to the reference MRL and control intensity as indicators of compliance or not.

**Fig. 5:**
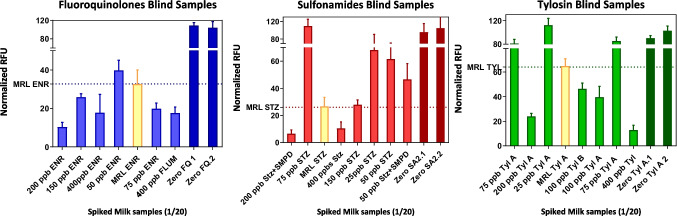
Pre-validation studies. Commercial milk samples were blindly doped at different levels above and below MRL for each analyte and randomly distributed on the array. Semi-quantitative data was obtained from the analysis after assay performance, detecting 18 from 20 samples correctly and without interfering with other analytes

A total of eighteen of twenty (18/20) milk samples were correctly identified, by referencing the signal obtained in the suspected sample with the signal measured in the reference MRL spiked positive control for each analyte respectively as displayed in Fig. 5. In fact, one false positive sample was detected for fluoroquinolones and this was not assumed as a limitation because every suspected positive sample from a screening method should always undergo a confirmatory analysis in order to validate the results. On the other hand, a false negative result was observed in a sample doped with TYLA, assuming that this could imply a potential risk if it is not properly controlled. Additionally, some milk samples were doped with two structural analogue antibiotics (sulfathiazole + sulfametoxypiridazine) showing that the additive contribution to the detectability was also evidenced by the class-selective reagents. More studies have to be performed to ensure the decision capabilities of this technology. Previous studies using the corresponding polyclonal antibodies [18, 34, 35], CCα and CCβ, were calculated and validated accordingly.

## Conclusions

Through the development of the already described DDI array, the optimization and characterization of several parameters were considered. In the first place, the assessment of the immunoreagents with a reference technique like ELISA, in buffer and milk reaching adequate sensitivity, was followed by the kinetic evaluation of the MAbs through SPR, suggesting that MAb-Tyl presented the highest affinity. Two distinct bioconjugation strategies were then established for covalent attachment of the hapten molecules with the oligonucleotide strands, following a proper characterization protocol.

Once the conjugates and antibodies were successfully evaluated, the DNA-directed immobilization approach under individual and multiplexed format was successfully demonstrated towards the detection of three reference antibiotic residues with LODs in the 1–5 ppb (µg kg^−1^) range, achieving comparable analytical performance with ELISA. Furthermore, the multiplexed DDI fluorescent array showed outstanding detectability reaching LODs of 0.89 µg kg^−1^ for CIP, 1.67 µg kg^−1^ for STZ, and 1.43 µg kg^−1^ for TYLA after a 20-fold milk dilution and without sample pretreatment. Furthermore, the platform also allowed simultaneous identification of the family of compounds detected by class antibodies included in the assay.

Even though, the detection of small molecules with a similar DDI approach has been demonstrated in our group towards anabolic androgenic steroids [32]. This is the first time, that antibiotic residue detection was achieved following an indirect competitive DDI approach in multiplexed configuration over milk samples. The high specificity of the class-selective antibodies allowed the determination of 18 different analytes in a single sample, without cross-reacting with other species. Lack of shared interactions between oligonucleotide strands was demonstrated to exploit multiplexed configuration. With this system, 23 samples can be determined in parallel with a minimum volume of 5 μL of milk.

Nowadays emerging nanotechnological approaches have been successfully applied in personalized diagnostics, food safety, and environmental monitoring. This work shows the potential of DNA-directed strategies as versatile and universal solutions for multiplexed screening tools in the food safety field. Our platform could be implemented to increase the number of samples screened against the presence of antibiotic residues, reducing costs associated with more complex analysis and offering an easy to use alternative in the real field. In addition, if the same oligonucleotides are conjugated to a different biomolecule, the already developed chip can be used to detect other analytes extending the number of applications available. Due to this, we encourage the use of this platform as screening tool for rapid and affordable analytical determination of antibiotics during milk production and processing chain. Thus, this technology can improve the high-throughput capabilities to address the high number of milk samples that has to be analyzed, as well as the implementation of DDI which can be affordable in portable devices as rapid test [46].

## Supplementary Information

Below is the link to the electronic supplementary material.

ESM 1 (DOCX 388 KB) 
