# Supplementary material for: DNA-directed immobilization fluorescent immunoarray for multiplexed antibiotic residue determination in milk
Source: Anal Bioanal Chem. 2024 Aug 28;416(30):7325–36. doi: 10.1007/s00216-024-05481-9 (PMC11584450; doi:10.1007/s00216-024-05481-9)

**Supporting Information**

**DNA directed immobilization fluorescent immunoarray for multiplexed antibiotic residues determination in milk**

**J. Guercetti^1,2^, N. Pascual^,1,2^, A. Aviñó^2,3^, R. Eritja^2,3^, J-P. Salvador^1,2^ and M.-P. Marco^1,2^**

1 Nanobiotechnology for diagnostics (Nb4D), Department of Chemical and Biomolecular Nanotechnology, Institute for Advanced Chemistry of Catalonia (IQAC) of the Spanish Council for Scientific Research (CSIC). Jordi Girona 18-26,08034, Barcelona, Spain.

2 CIBER de Bioingeniería, Biomateriales y Nanomedicina (CIBER-BBN),

3 Nucleic acid chemistry group, Department of Chemical and Biomolecular Nanotechnology, Institute of advanced chemistry of Catalonia (IQAC), Spanish National Research Council (CSIC), Jordi Girona 18-26, 08034, Barcelona, Spain.

*Corresponding Author:

Dr. J.-Pablo Salvador

Nanobiotechnology for diagnostics group

CIBER-BBN/IQAC-CSIC

Jordi Girona, 18-26

08034-Barcelona

Spain

Phone: +34 93 4006100, Ext 437892

E-mail: jpablo.salvador@iqac.csic.es

**1. Characterization of immunoreagents by ELISA**

- 1. **ELISA assays for FQ, SA and Tyl**

Haptenized BSA conjugates (PrEDA, SA2 and hTB) and antibodies (MAb-FQ, MAb-Tyl, MAb-SA) were characterized through indirect competitive ELISA to asses sensitivity, cross reactivity and matrix effect towards the target antibiotic detection prior their implementation in the microarray format. Individual assays were performed for each reference target analyte (SPY, TYLA and CIP) in PBST Ca^2+^ and milk (1/5) diluted in PBST Ca^2+^. 96- well plates NUNC MaxiSorp (Thermo Fisher Scientific, USA) were coated with PrEDA-BSA (0.5 μg mL^-1^), hTB-BSA (0.0625 μg mL^-1^) and SA2-BSA (0.125 μg mL^-1^) in coating buffer 100 μl/well and incubated overnight 4^o^ C. The following day, the microplates were washed four times 300 µL/well with PBST using plate washer BioTek 405 LS (Agilent, USA). Then, standard calibration curves ranging from 5 µM to 0.5 nM were prepared in PBST-Ca^2+^ and in two milk sample dilutions at 1/5 and 1/20 in PBST-Ca^2+^. For the case of individual assays, a single antibody was used and for studies of shared reactivity the “cocktail of antibodies” was required (maintaining the same concentrations in both formats: MAb-FQ 0.125 μg mL^-1^, MAb-Tyl 0.0156 μg mL^-1^ ,MAb-SA 0.0625 μg mL^-1^) for 30 minutes incubation. After a washing procedure, 100 µl of a solution of anti-mouse IgG HRP (1/6000) in 10 mM PBST was incubated for 30 min. The next step consisted on the addition 100 μl of the substrate solution (citrate solution, H_2_O_2_ 0.0004 % and TMB 0. 375 μg mL^-1^) covered from light additional 30 mins. Finally, 50 μl of 4M H_2_SO_4_ per well was used to stop colorimetric reaction on each well and absorbance was measured at 450 nm. The standard curves were fitted to a four-parameter equation according to the formula: Y = [(A - B)/1 - (x/C) D̂] + B, where A is the maximal absorbance, B is the minimum absorbance, C is the concentration producing 50 % of the maximal absorbance, and D is the slope at the inflection point of the sigmoid curve.

**Matrix effect of respective immunoreagents in ELISA**

*Figure S1. Matrix effect of ERFX, SPY and TYLA detection through indirect competitive ELISA. Individual*

|  |  |
| --- | --- |
|  |  |

Table S1 Analytical parameters for FQ’s ELISA at different milk dilutions

| **ERFX** | **PBST Ca^2+^** | **Undiluted** | **Milk 1/2** | **Milk 1/5** | **Milk 1/10** | **Milk 1/20** |
| --- | --- | --- | --- | --- | --- | --- |
| Amax | 2.05 | 1.44 | 1.73 | 2.25 | 2.43 | 2.42 |
| Amin | 0.01 | 0.01 | 0.02 | 0.03 | 0.04 | 0.04 |
| Slope | -0.95 | -0.86 | -0.96 | -0.98 | -0.99 | -0.94 |
| IC50a | 3.83±0.03 | 2.47±0.05 | 1.95±0.04 | 2.71±0.01 | 2.20±0.03 | 3.73±0.03 |
| R^2^ | 0.99 | 0.99 | 0.99 | 0.99 | 0.99 | 0.99 |

*^a^* Concentrations expressed in μg L^-1^ . On each assay the standard curve was build using three well-replicates.

Table S2 Analytical parameters for SA’s ELISA at different milk dilutions

| **SPY** | **PBST Ca^2+^** | **Undiluted** | **Milk 1/2** | **Milk 1/5** | **Milk 1/10** | **Milk 1/20** |
| --- | --- | --- | --- | --- | --- | --- |
| Amax | 1.96 | 0.98 | 1.12 | 1.35 | 1.29 | 1.36 |
| Amin | 0.11 | 0.04 | 0.04 | 0.03 | 0.05 | 0.04 |
| Slope | -1.89 | -1.212 | -1.27 | -1.32 | -1.71 | -1.72 |
| IC50a | 5.56±0.08 | 0.93±0.04 | 0.88±0.02 | 1.31±0.02 | 0.75±0.02 | 2.48±0.02 |
| R^2^ | 0.99 | 0.98 | 0.99 | 0.99 | 0.99 | 0.99 |

*^a^* Concentrations in μg L^-1^. On each assay the standard curve was build using three well-replicates.

Table S3 Analytical parameters for TYL’s ELISA at different milk dilutions

| **TYLB** | **PBST Ca^2+^** | **Undiluted** | **Milk 1/2** | **Milk 1/5** | **Milk 1/10** | **Milk 1/20** |
| --- | --- | --- | --- | --- | --- | --- |
| Amax | 0.93 | 0.41 | 0.43 | 0.60 | 0.74 | 0.72 |
| Amin | 0.05 | 0.05 | 0.04 | 0.05 | 0.04 | 0.04 |
| Slope | -0.75 | -0.82 | -0.92 | -0.84 | -0.61 | -0.73 |
| IC50a | 2.78±0.06 | 2.73±0.03 | 2.80±0.02 | 3.45±0.05 | 1.48±0.02 | 3.61±0.06 |
| R^2^ | 0.99 | 0.99 | 0.99 | 0.99 | 0.99 | 0.99 |

^a^ Concentrations in μg. L^-1^ .. On each assay the standard curve was build using three well-replicates.

Table S4 Immunoreagents utilized in the development of the immunochemical platforms

| **Target Analytes** | **MAb’s** | **Clon N^o^** | **Bioconjugate** | **Hapten** |
| --- | --- | --- | --- | --- |
| FQ’s | MAb-FQ | FQ1109C2.2 | PrEDA-BSA | PrEDA |
| SA’s | MAb-SA | 8E5-16085E | SA2-BSA | SA2 |
| TYL’s | MAb-Tyl | C6.23.4.2 | hTB-BSA | hTB |

**2. Kinetic characterization of monoclonal antibodies and hapten-BSA conjugates through SPR**

**SPR characterization Biacore T200**

Kinetic characterization of immunoreagents was carried out through Surface Plasmon Resonance (SPR) technique using Biacore T200 system. Initial pH scouting experiments were assessed in order to define the most suitable immobilization condition using 10 mM sodium acetate pH 4 to couple BSA conjugates to carboxymethylated gold sensor chips. Afterwards, single cycle kinetic experiments were conducted by consecutive injections of increasing concentrations of selective antibodies with regeneration steps using Glycine-HCl 0.1M for suflonamides and fluoroquinolones reagents and 10mM NaOH for tylosin assay. The processed sensograms for each immunoreagents are represented in the following section indicating the estimated kinetic parameters determined with the Biacore T200 analysis software Gold sensor chip preparation

A standard amine coupling procedure was applied at 25^o^C to immobilize the BSA-hapten conjugates (BSA-PrEDA, BSA-SA2, BSA-hTB) over a sensor chip CM5 series S (Cytiva, USA). For an adequate immobilization, the BSA-hTB conjugate was diluted to 10 μg mL^-1^in 10 mM sodium acetate at different pH’s and tested using the pH scouting wizard in the Biacore T200 control software (GE Healthcare). A better immobilization level was archived using sodium acetate pH 4 and due to this was employed as the immobilization buffer in the following procedures for all BSA conjugates.

The carboxylmethylated (CM) gold sensor chip was separated in four microfluidic channels utilized for the immobilization of the three bioconjugates while the remaining channel was used as reference for non-specific binding immobilizing only BSA. Surface activation took place after injecting 150 µL of a mixture of EDC and NHS (1:1 *v*/*v*) at a flow rate of 15 µL/min. Then, BSA conjugates diluted (25 μg mL^-1^) in immobilization buffer (10 mM sodium acetate, pH 4) were injected in flow cells 2 to 4 respectively (channel 2: BSA-PrEDA, channel 3: BSA-SA2, channel 4: BSA-hTB). Flow cell 1 was used as a reference channel for non-specific binding injecting 25ug/ml of BSA. The immobilization protocol concluded with the addition of 1M ethanolamine to block potential reactive groups over CM-dextran surface. The immobilization level achieved over each channel expressed in resonance units (RU’s) was, channel 1 BSA=166 RU’s, channel 2 BSA-PrEDA= 304 RU’s, channel 3 BSA-SA2=71 RU’s, channel 4 BSA-hTB=236 RU’s. Although, relatively low RU’s levels are preferred for kinetics determinations considering the mass of the ligands, the analyte and the signal expected theoretical immobilization levels were estimated through the determination of target immobilization (RL) using the following formula, Rmax = RL x MW_analyte_/MW_ligand_ x Sm. (Rmax = theoretical max response for analyte binding 100. Sm = stoichiometry of Analyte/Ligand. RL = RU of immobilized ligand (target density).

All kinetic determinations were performed in freshly prepared, filtered and degassed running buffer containing 10 mM PBST and 1mM CaCl_2_ at 25°C. The stock aliquots of respective antibodies (13.3 µM) were first diluted 1/40 and from that, serial dilutions 1/2 in running buffer were prepared to define the working range (V_f_=400 µL). Single cycle kinetic experiments were performed, starting with three injections at with running buffer to define a stable baseline. Then serial injections of 30 µL of antibody solution with 120 seconds for association and 300 seconds dissociation time at a flow rate 30 µL/min were performed. Afterwards, surface regeneration was carried out with 2 pulses of 5 µL of 0.1 M glycine-HCl, pH 2.7 for BSA-SA2 and BSA-PrEDA conjugates, but in the case of BSA-hTB conjugate the regeneration solution was 1 mM NaOH, requiring more aggressive conditions for complete removal of antibodies.

The Biacore T200 Evaluation Software (v 2.02) was used to estimate equilibrium dissociation constants, off-rates and on-rates values determined from the analysis of the experimental sensograms obtained with Biacore system, fitting a 1:1 Langmuir binding model while considering mass transfer limitation.

An efficient immobilization procedure was implemented defining a low coating density to avoid steric hindrance or aggregation, reduce mass transport limitation and also to obtain a higher range of controlled binding {Pol, 2016 #62}. Based on the results obtained during the pH scouting, sodium acetate pH 4 was selected as immobilization buffer to favor protein accumulation over carboxymethylated surface. Then single cycle kinetic experiments were performed to determine the level of interaction and affinity constants of BSA-hapten conjugates with the monoclonal antibodies in running buffer obtaining dose dependent response.

Regarding association and dissociation rates estimation, MAb-FQ showed fast association rate (10^-4^) and relatively low dissociation rates. While for MAb-SA and MAb-Tyl a similar association rate (10^-5^) was determined but dissociation occurred differently for both cases. On one hand, a fraction of MAb-SA2 was easily unbound during the dissociation phase without addition of regeneration buffer indicating weaker binding compared to MAb-Tyl that remained complexed with the hapten conjugate during the same phase. In addition, MAb-Tyl required a more restrictive regeneration solution compared to the rest of antibodies removed with standard 0.1M Glycine-HCl, suggesting that a stronger interaction was taking place as a sign of the low dissociation rate measured.

Furthermore, the assay configuration immobilizing the bioconjugates over the gold surface allowed the determination of the affinity constant between the antibodies and the hapten molecules.

| 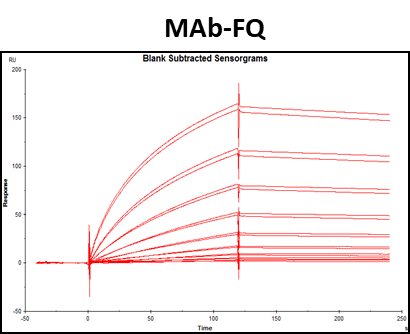 | 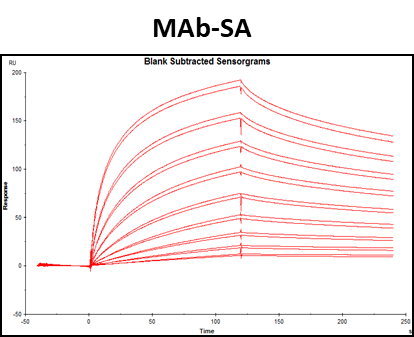 |
| --- | --- |
| 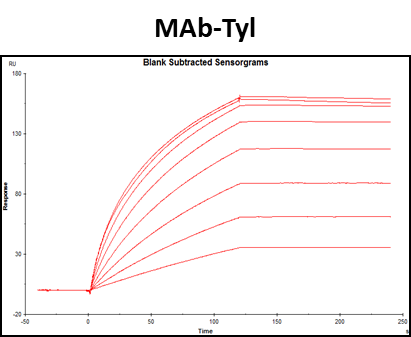 |  |

*Figure S2. Kinetic characterization Ag/Ab complex MAb-FQ/BSA-PrEDA, MAb-SA/BSA-SA2 and MAb-TYL/BSA-hTB with Biacore T200. Duplicate injections of 9 antibody dilutions in PBST Ca^2+^ ranging from 332nM-0.64nM were assessed to define kinetic parameters. Biacore T200 software evaluation 2.0 was employed to process raw sensogram.*

Table S5- Kinetic parameters of Ag/MAb pairs assessed in Biacore T200. It shows association rate constant (Ka), dissociation rate constant (Kd), and binding constant (KD) of antibodies with respective BSA-hapten conjugates. The analysis was performed through Biacore T200 evaluation software, version 2.0 to determine Ka and Kd using 1:1 fitting model. Ms, millisecond; M, molar; s, second.

| Kinetic constants (N=1) | **MAb-FQ** | **MAb-SA** | **MAb-Tyl** |
| --- | --- | --- | --- |
| ka (1/Ms) | 6.424x10^+4^ | 2.505x10^+5^ | 3.166x10^+5^ |
| kd (1/s) | 4.590x10^-4^ | 0.002752 | 4.328x10^-5^ |
| KD (M) | 7.14610^-9^ | 1.098x10^-8^ | 1.367x10^-10^ |

**3. Matrix effect of single-analyte DDI microarrays in cow’s milk dilution**

|  |  |  |
| --- | --- | --- |

*Figure S3. Matrix effect of milk 1/5 in the DDI microarray. The graphs show the calibration curves in buffer and 1/5 diluted milk (10mM PBST Ca^2+^) following an indirect competitive DDI format. Each data point is the average and standard deviation of 10 spot replicates of two microarray chips performed in two different days.*

Based on the matrix effect performed in ELISA, the assessment of milk in the DDI array was performed by building standard curves (5 μM to 0.5 nM) in PBST Ca^2+^and in milk diluted 1/5 in the same buffer. Both curves were measured with the DDI microarray approach following the standard assay procedure and results are expressed in the figure S2. A similar detectability was achieved under both conditions for CIP assay, while for SPY and TYLA assay the IC50 was slightly improved in diluted milk. Only in the case of CIP the RFU_max_ was lower in milk 1/5, and this can be attributed to the blocking effect produced by a complex matrix as milk.

**4. Cooperative phenomena of the monoclonal antibody used**

*Figure S4. The shared reactivity between all immunoreagents used. Image A) the cocktail of oligonucleotides (N4down/N4up-PrEDA, N5down/N5up-SA2 and N6down/N6up-TylB) used against the specific antibody separately. Also, it is showed the recognition between the cocktail of oligonucleotide against the cocktail of all MAbs (Image B). Finally, Image C shows the specific recognition of the cocktail of oligonucleotides, plus the cocktail of MAbs in the presence of ciprofloxacin (Cipro as FQ compound), sulfapyridine (SPyr as SA compound) and Tylosine A (TylA as tylosine*)


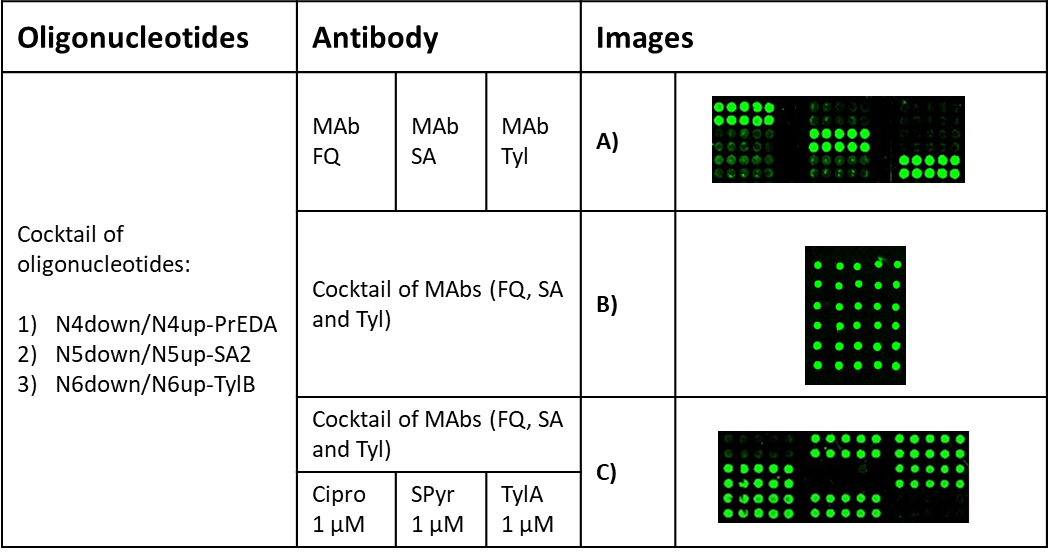

Supplement: Supplementary file 1 — (DOCX 388 KB) [file 216_2024_5481_MOESM1_ESM.docx]
